# Head-to-head comparison of two engineered cardiac grafts for myocardial repair: From scaffold characterization to pre-clinical testing

**DOI:** 10.1038/s41598-018-25115-2

**Published:** 2018-04-30

**Authors:** Isaac Perea-Gil, Carolina Gálvez-Montón, Cristina Prat-Vidal, Ignasi Jorba, Cristina Segú-Vergés, Santiago Roura, Carolina Soler-Botija, Oriol Iborra-Egea, Elena Revuelta-López, Marco A. Fernández, Ramon Farré, Daniel Navajas, Antoni Bayes-Genis

**Affiliations:** 1 ICREC Research Program, Health Science Research Institute Germans Trias i Pujol, Badalona, Spain; 2 CIBER de Enfermedades Cardiovasculares, Madrid, Spain; 3 Centre of Regenerative Medicine in Barcelona, Barcelona, Spain; 4 Biophysics and Bioengineering Unit, Faculty of Medicine and Health Sciences, University of Barcelona, Barcelona, Spain; 5 Institute for Bioengineering of Catalonia, The Barcelona Institute of Science and Technology, Barcelona, Spain; 6 CIBER de Enfermedades Respiratorias, Madrid, Spain; 7 Anaxomics Biotech, Barcelona, Spain; 8 Flow Cytometry Facility, Germans Trias i Pujol Research Institute, Campus Can Ruti, Badalona, Spain; 9 Institut d’Investigacions Biomèdiques August Pi i Sunyer, Barcelona, Spain; 10 Cardiology Service, Germans Trias i Pujol University Hospital, Badalona, Spain; 11 Department of Medicine, Universitat Autònoma de Barcelona, Bellaterra, Spain

## Abstract

Cardiac tissue engineering, which combines cells and supportive scaffolds, is an emerging treatment for restoring cardiac function after myocardial infarction (MI), although, the optimal construct remains a challenge. We developed two engineered cardiac grafts, based on decellularized scaffolds from myocardial and pericardial tissues and repopulated them with adipose tissue mesenchymal stem cells (ATMSCs). The structure, macromechanical and micromechanical scaffold properties were preserved upon the decellularization and recellularization processes, except for recellularized myocardium micromechanics that was ∼2-fold stiffer than native tissue and decellularized scaffolds. Proteome characterization of the two acellular matrices showed enrichment of matrisome proteins and major cardiac extracellular matrix components, considerably higher for the recellularized pericardium. Moreover, the pericardial scaffold demonstrated better cell penetrance and retention, as well as a bigger pore size. Both engineered cardiac grafts were further evaluated in pre-clinical MI swine models. Forty days after graft implantation, swine treated with the engineered cardiac grafts showed significant ventricular function recovery. Irrespective of the scaffold origin or cell recolonization, all scaffolds integrated with the underlying myocardium and showed signs of neovascularization and nerve sprouting. Collectively, engineered cardiac grafts -with pericardial or myocardial scaffolds- were effective in restoring cardiac function post-MI, and pericardial scaffolds showed better structural integrity and recolonization capability.

## Introduction

Cardiac tissue engineering, which combines the use of cells and biomaterials, has been proposed as an alternative therapy for myocardial infarction (MI)^1^, with the goals of repairing the damaged myocardium, recovering heart function, and preventing ventricular remodeling in end-stage heart failure. In tissue engineering, cells are embedded within a natural or synthetic scaffold, avoiding the harsh infarcted milieu, and improving the low cell retention and survival reported for direct cell injection^2,3^. Accordingly, the scaffold biomaterial is crucial, and ideally should closely resemble the physiological myocardial extracellular matrix (ECM) properties as internal scaffold conformation, mechanics, and composition will modulate cellular differentiation, migration, and adhesion^4–8^.

In this context, decellularized cardiac tissues provide a close match to the native, physiological microenvironment, as they preserve the inherent stiffness, composition, vasculature network, and three-dimensional framework^9^, and enable electromechanical coupling with the host myocardium upon implantation^10,11^. Herein, we tested two decellularized scaffolds generated from the myocardium and pericardium, as previously described^12,13^. The scaffolds were either repopulated with porcine adipose tissue mesenchymal stem cells (pATMSCs) or tested as cell-free scaffolds. Macro and micromechanics, structural characteristics and protein content of the decellularized scaffolds were evaluated prior to recellularization. The functional benefits associated with both the acellular and recellularized scaffolds were assessed in a preclinical MI swine model. Cardiac function was analyzed with magnetic resonance imaging (MRI), and accurate infarct size was obtained with late gadolinium enhancement.

## Methods

### Generation of decellularized myocardial and pericardial scaffolds

Myocardial tissue samples were isolated from porcine hearts (n = 22), and surgical samples of pericardial tissue were acquired from 90 patients (63 males, 27 females; mean age 69 ± 10 years; range 42 to 85 years) undergoing cardiac interventions at our institution, with apparently healthy pericardia and after signed consent. This study was revised and approved by the Germans Trias i Pujol University Hospital ethics committee, and all the protocols conformed to the principles outlined in the Declaration of Helsinki. Both cardiac tissues were decellularized, lyophilized, and sterilized as described elsewhere^12,13^.

### Recellularization of sterilized cardiac scaffolds

The sterilized acellular cardiac scaffolds were repopulated with pATMSCs obtained from porcine pericardial adipose tissue, as previously reported^13^, to generate both the engineered myocardial and pericardial grafts. Initially, the decellularized scaffolds were rehydrated with a mixture of 175 μL of peptide hydrogel RAD16-I (Corning, Corning, NY) and 175 μL of GFP^+^-pATMSCs (1.75 × 10^6^) in 10% sucrose (Sigma-Aldrich, St Louis, MO), added dropwise on top of the scaffold. To promote hydrogel jellification, α-minimum essential medium eagle (α-MEM) (Sigma-Aldrich) was added over the scaffold. The produced cell-enriched grafts were maintained over one week under standard culture conditions (37 °C, 95% air, 5% CO_2_), changing medium every 2 days.

### Flow cytometry

Changes in cell viability were assessed by flow cytometry using the Annexin V Apoptosis Detection Kit and the 7-amino-actinomycin D (7-AAD) viability staining solution (eBiosciences). Cells (2–5 × 10^5^) were labelled according to the supplier’s instructions. The percentage of viable cells was measured by using a Coulter EPICS XL flow cytometer with the Expo32 software (Beckman Coulter).

### Measurement of micromechanical properties with atomic force microscopy (AFM)

Micromechanical properties of native myocardial tissue and decellularized and recellularized scaffolds were studied in 5 porcine hearts. 50 µm thick slices of fresh myocardium were obtained using a vibratome (0.01 mm/s blade velocity; 2 mm amplitude; VT 1200 S, LEICA, Germany) and attached to glutaraldehyde pre-treated glass slides and directly measured with atomic force microscopy (AFM). The decellularized and recellularized myocardial scaffolds (15 × 5 × 5 mm) were embedded in 3:1 (v:v) solution of optimal cutting temperature medium (OCT, TissueTek, USA) in phosphate buffered saline (PBS) 1x and frozen at −80 °C. For AFM measurements, 50 µm thick scaffold slices were cut using a cryostat (HM 560 CryoStar, Thermo Fisher, Madrid, Spain), placed on top of positively charged glass slides and stored at −20 °C until AFM measurements. To assess the effect of the different steps of the recellularization process in tissue stiffness, AFM measurements were performed in myocardial samples after lyophilization, sterilization and peptide hydrogel embedding (5 additional porcine hearts). A similar AFM measurement protocol was performed to measure the stiffness of human pericardial samples but in this case native pericardial stiffness was measured in 5 × 5 × 2 mm samples adhered with cyanoacrylate to a petri dish.

Before AFM measurements, the slices with OCT were rinsed several times with PBS until OCT was completely removed. Then, the slides were placed on the sample holder of a custom-built AFM system^12^ attached to an inverted optical microscope (TE 2000, Nikon, Japan), and force-indentation (*F* − *δ*) curves were measured at RT using cantilevers with a polystyrene microsphere 4.5 μm in diameter attached at its end (nominal spring constant of 0.03 N/m, Novascan, Ames, IA). The actual spring constant of the cantilevers was calibrated with the thermal noise method. The micromechanical Young’s modulus (*E*_*m*_) was computed by fitting *F* − *δ* data with the Hertz contact model^12^

1 $$F=\frac{4\cdot {E}_{m}\cdot {R}^{1/2}}{3(1-{v}^{2})}{\delta }^{3/2}$$

where *R* is the radius of the microsphere and *ν* the Poisson’s ratio (assumed to be 0.5).

For each slice, measurements were performed in two locations separated by ~500 µm. At each location, five measurements were taken separated by ~10 µm following a linear pattern. At each measurement point, five *F* − *δ* force curves with a maximum indentation of 1 µm were recorded. Myocardium and pericardium micromechanical stiffness was characterized as the average *E*_*m*_ computed from the different force curves recorded in each sample.

### Measurement of macromechanical properties with tensile testing

For macromechanical measurements, strips (~8 × 1 × 1 mm) of native, decellularized, recellularized, lyophilized, sterilized and hydrogel embedded myocardium and pericardium cut along the long-axis with a scalpel were used (n = 5 each). The strips were maintained at RT in Krebs-Henseleit solution (Sigma-Aldrich). For tensile testing, each strip was gently dried with tissue paper and its mass (*M*) measured. The unstretched length (*L*_0_) of the strip was measured and its cross-sectional area (*A*) computed as

2 $$A=\frac{M}{\rho \cdot {L}_{o}}$$

where *ρ* is the tissue density (assumed to be 1 g/cm^3^). One end of the strip was glued with cyanoacrylate to a hook attached to the lever of a servocontrolled displacement actuator, which simultaneously stretched the strip and measured the stretched length (*L*) and the applied force (*F*) (300C-LR, Aurora Scientific, Ontario, Canada). The other end of the strip was glued to a fixed hook. Measurements were performed inside a bath with Krebs-Henseleit solution at 37 °C. The stress (*σ*) applied to the strip was defined as:

3 $$\sigma =\frac{F}{A}$$

Tissue stretch (*λ*) was defined as:

4 $$\lambda =\frac{L}{{L}_{0}}$$

Strips were initially preconditioned by applying 10 cycles of cyclic stretching at a frequency of 0.2 Hz and maximum stretch of ∼50%. *L*_0_ was measured again and *F* − *L* data from 10 more cycles were recorded. The stiffness of the tissue was characterized by the macromechanical Young’s modulus (*E*_*M*_) defined as

5 $${E}_{M}=\frac{{\rm{d}}\sigma }{{\rm{d}}\lambda }$$

Stress-stretch (*σ* − *λ*) curves were analyzed with Fung’s model, which assumes that *E*_*M*_ increases linearly with stress as:

6 $${E}_{M}=\alpha \cdot (\sigma +\beta )$$

where *α*·*β* is the value of *E*_*M*_ at *L*_0_ and *α* characterizes the strain-hardening behavior of the tissue. Eq. 6 leads to a stress-stretch exponential dependence

7 $$\sigma =({\alpha }_{r}+\beta ){e}^{\alpha (\lambda -{\lambda }_{r})}-\beta $$

where *σ*_*r*_ and *λ*_*r*_ define an arbitrary point of the *σ* − *λ* curve. The parameters of Eq. 7 were computed for each curve by non-linear least-squares fitting using custom built code (MATLAB, The MathWorks, Natick, MA). Using these parameters, *E*_*M*_ was computed at unstretched length (*λ* = 1) and at 20% stretch (*λ* = 1.2). Macromechanical stiffness of each heart strip was characterized as the average *E*_*M*_ computed from the last *σ* − *λ* curves recorded.

### Proteomic analysis

The proteins from decellularized tissues were initially extracted and digested as detailed in the *Supplementary methods*. Peptides were analyzed in an Impact II Q-TOF mass spectrometer (Bruker, Billerica, MA) using the nano-LC dedicated CaptiveSpray source, coupled to a nanoRSLC ultimate 3000 system (Thermo Fisher). The peptide separation details are also found in the *Supplementary methods*.

The mass spectrometer was operated in the Instant Expertise mode, consisting of a cycle of: (i) One MS spectrum in 250 msec, mass range 150–2200 Da; (ii) as many as possible MS/MS spectra in 1.75 sec, at 32–250 msec, depending on precursor intensity, mass range 150–2200 Da; and (iii) precursor exclusion after one selection. Searches against the Swissprot (2015–08) library of mammal protein sequences were performed using the Mascot 2.5 search engine (MatrixScience, Boston, MA), with a 7 ppm tolerance at the MS level and 0.01 Da tolerance at MS/MS level, and a false discovery rate fixed to a maximum value of 1%. The variable modifications were carbamidomethyl, Gln->pyro-Glu, acetyl, oxidation, and deamidation.

### Animal experiment design

All animal studies were approved by the Minimally Invasive Surgery Centre Jesús Usón Animal Experimentation Unit Ethical Committee (Number: ES 100370001499) and complied with all the guidelines concerning the use of animals in research and teaching, as defined by the Guide for the Care and Use of Laboratory Animals (NIH Publication No. 80-23, revised 1996). Seventy-four crossbred Landrace × Large White juvenile pigs (30.6 ± 5.5 kg) were submitted to a MI and randomly distributed into five groups as follows:

1. Control MI (n = 17): no scaffold implantation;
2. Per-MI (n = 17): cell-free pericardial scaffold implantation;
3. Myo-MI (n = 8): cell-free myocardial scaffold implantation;
4. Per-ATMSCs (n = 22): pATMSC-enriched pericardial scaffold implantation;
5. Myo-ATMSCs (n = 10): pATMSC-enriched myocardial scaffold implantation.

After a left lateral thoracotomy, MI was induced by double ligation of the first marginal branch of the circumflex artery, as previously described^14^. After 30 min, the scaffolds were attached over the infarcted area with 0.1–0.2 mL of surgical glue (Glubran®2, Cardiolink, Barcelona, Spain). After recovery, the animals were housed for 40 days before sacrifice.

### Cardiac Function Assessment

Cardiac MRI was performed at 1.5 T (Intera, Philips, Amsterdam, the Netherlands) in all animals using a four-channel phased array surface coil (SENSE Body Coil, Philips). A breath-held, ECG-gated cine steady-state precession MRI was acquired (TR/TE 4.1/2.1 ms; flip angle 60°; field of view 320 × 320 mm; matrix 160 × 160 pixels, slice thickness 7 mm; bandwidth 1249.7 Hz/pixel). Delayed enhancement images were acquired after intravenous gadolinium (Gd-DTPA, 0.2 mL/kg) using a phase-sensitive inversion recovery sequence (TR/TE 4.9/1.6 ms; flip angle 15°; inversion time 157 ms; field of view 330 × 330 mm; matrix 224 × 200 pixels; slice thickness 10 mm, bandwidth 282.3 Hz/pixel). The left ventricular ejection fraction (LVEF), left ventricular end-systolic volume (LVESV), and left ventricular end-diastolic volume (LVEDV) were measured at baseline, 48 h after MI, and before sacrifice. Independent blinded investigators carried out the MRI data acquisition and analysis.

### Morphometric analysis

Sacrifices were performed at 39.3 ± 7.5 days after MI with an intravenous injection of potassium chloride solution. Following a lateral thoracotomy, porcine hearts were excised. The left ventricular (LV) infarct area was measured in photographed heart sections obtained 1.5 cm distal to the artery ligation, as previously described^14^. Double-blind quantification was performed by two independent investigators using Image J software (version 1.48, National Institutes of Health, Bethesda, MD).

### Histopathological and immunohistological analysis

Paraffin slices (4-μm) were stained with hematoxylin/eosin (H/E), Movat’s pentachrome, Movat’s modified (for simultaneous collagen and mucopolysaccharide acid staining^15^, and Gallego’s modified trichrome to analyze histological changes and scaffold recellularization using a computer-associated Olympus CKX41 microscope (Olympus, Tokyo, Japan) with a ProgRes® CF Cool camera (Jenoptik, Jena, Germany).

Frozen 10-μm sections were immunostained with biotinylated *Griffonia simplicifolia* lectin I B4 (IsoB4; 1:25; Vector Labs, Burlingame, CA), phalloidin-Atto 565 (1:50; Sigma-Aldrich), smooth muscle actin (SMA; 1:50; Sigma-Aldrich), type-I collagen (col-I) and type-III collagen (col-III) (1:100; Abcam, Cambridge, UK), cardiac troponin I (cTnI) (1:100; Abcam), cardiac troponin T (cTnT) (1:100; AbD Serotec), and elastin (1:100; Abcam) primary antibodies to characterize the scaffolds before and after their implantation. Secondary antibodies included Alexa Fluor 488-conjugated streptavidin (1:500; Molecular Probes, Eugene, OR), Cy2, Cy3, and Cy5 (1:500; Jackson ImmunoResearch Laboratories, West Grove, PA). All sections were counterstained with 4′,6-diamidino-2-phenylindole (DAPI) (0.1 µg/mL; Sigma-Aldrich) and analyzed by confocal microscopy (Axio-Observer Z1, Zeiss, Oberkochen, Germany).

### Scanning electron microscopy (SEM)

Native tissues and decellularized, recellularized scaffolds, and engineered grafts after implantation were first washed with sterile distilled water and fixed in 10% formalin (Sigma-Aldrich) O/N at 4 °C. Then, samples were washed with sterile distilled water, subsequently dehydrated in increasing concentrations of ethanol solutions, and preserved in absolute ethanol at 4 °C, until transference to a CO_2_ critical point dryer (EmiTech K850; Quorum Technologies, Lewes, UK). Finally, the scaffolds were sputter-coated with gold using an ion sputter (JFC 1100, JEOL, Tokyo, Japan) and examined with a JSM-6510 (JEOL) scanning electron microscope at 15 kV.

### Pore size measurements

The pore size of both decellularized myocardial and pericardial scaffolds (n = 3 of each) was blindly evaluated using 10 randomly selected SEM images with a magnification range from 500 to 1500, and at least 10 different pores from each image were randomly chosen and measured to generate an average value. For each pore, the long and short axis lengths were determined using ImageJ software, and pore roundness was calculated as the ratio between the short and long axis.

### Statistical analysis

Data are represented as the mean ± SEM. Statistical analyses were performed through the independent Student’s t test for cell number and density, pore analysis, and myocardium vs. pericardium mechanical analysis; paired sample t-test was used to analyze cell viability experiments and one-way analysis of variance (ANOVA) with the Tukey’s *post*-*hoc* correction for multiple comparisons was conducted for mechanical, MRI and infarct size data. SPSS 21.0.0.0 (SPSS, Inc, Chicago, IL) and SigmaPlot 13 (Systat Software Inc, San Jose, CA) were used for data analysis. Values were considered significant when *P* < 0.05 and tendency when *P* < 0.10.

The datasets generated and/or analysed during the current study are available from the corresponding author on reasonable request.

## Results

### Structural and mechanical characterization

**Figure 1:**
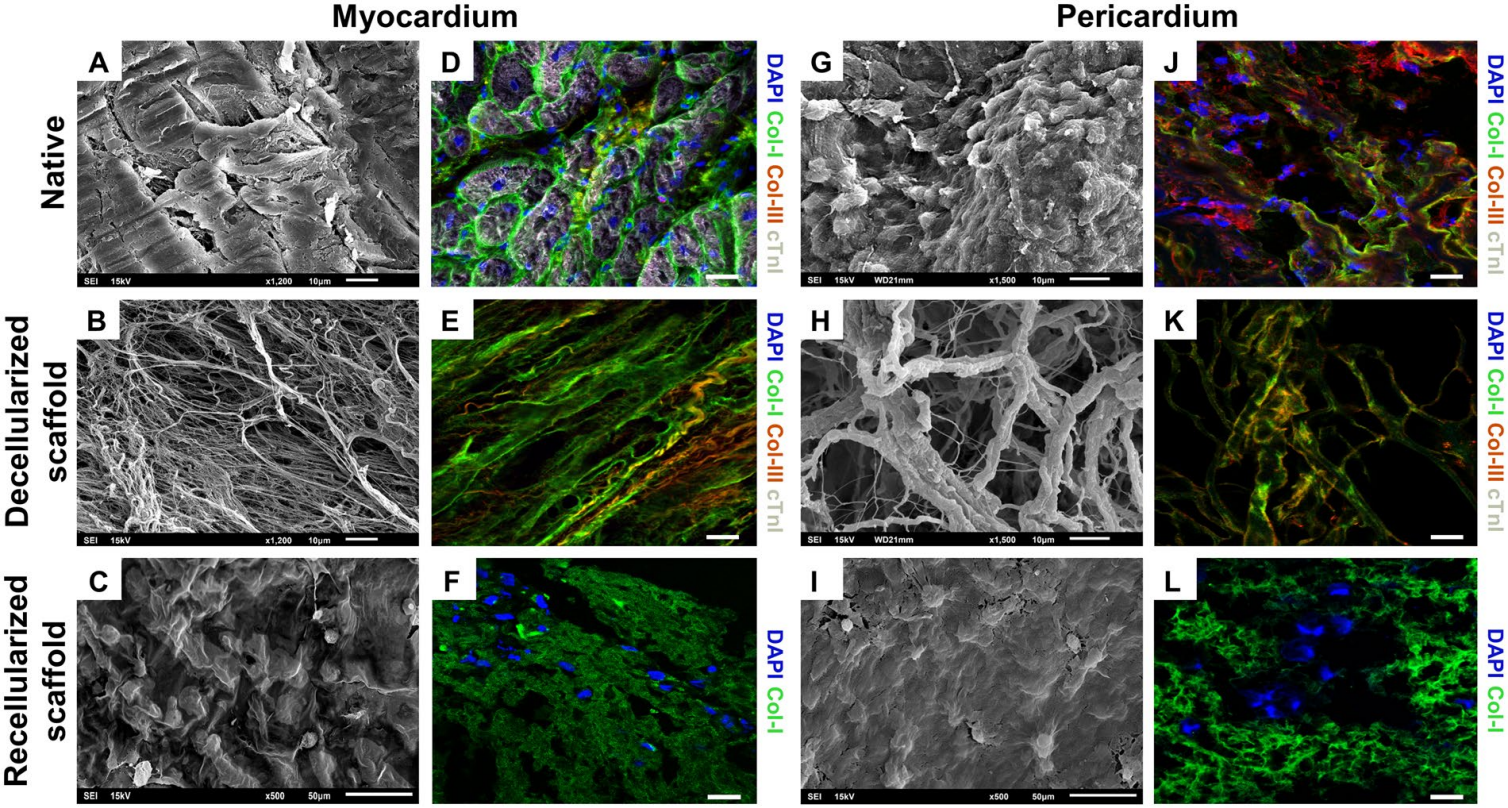
Internal structure and protein composition of the cardiac scaffolds. Ultrastructure determined by SEM of the (**A**) native myocardium and (**B**) acellular myocardial scaffolds; or (**G**) native pericardium and (**H**) acellular pericardial scaffolds. (**D**,**E**) Representative images for the native myocardium and decellularized myocardial scaffolds, respectively; and (**J**,**K**) native pericardium and decellularized pericardial scaffolds showing immunostaining for col-I (green), col-III (red), and cTnI (white). (**C**) SEM images for recellularized myocardial and (**I**) pericardial scaffolds. (**F**,**L**) Photographs displaying immunostaining for col-I (green) for recellularized myocardial and pericardial scaffolds. Nuclei were counterstained with DAPI (blue). Scale bars = 50 μm.

**Figure 2:**
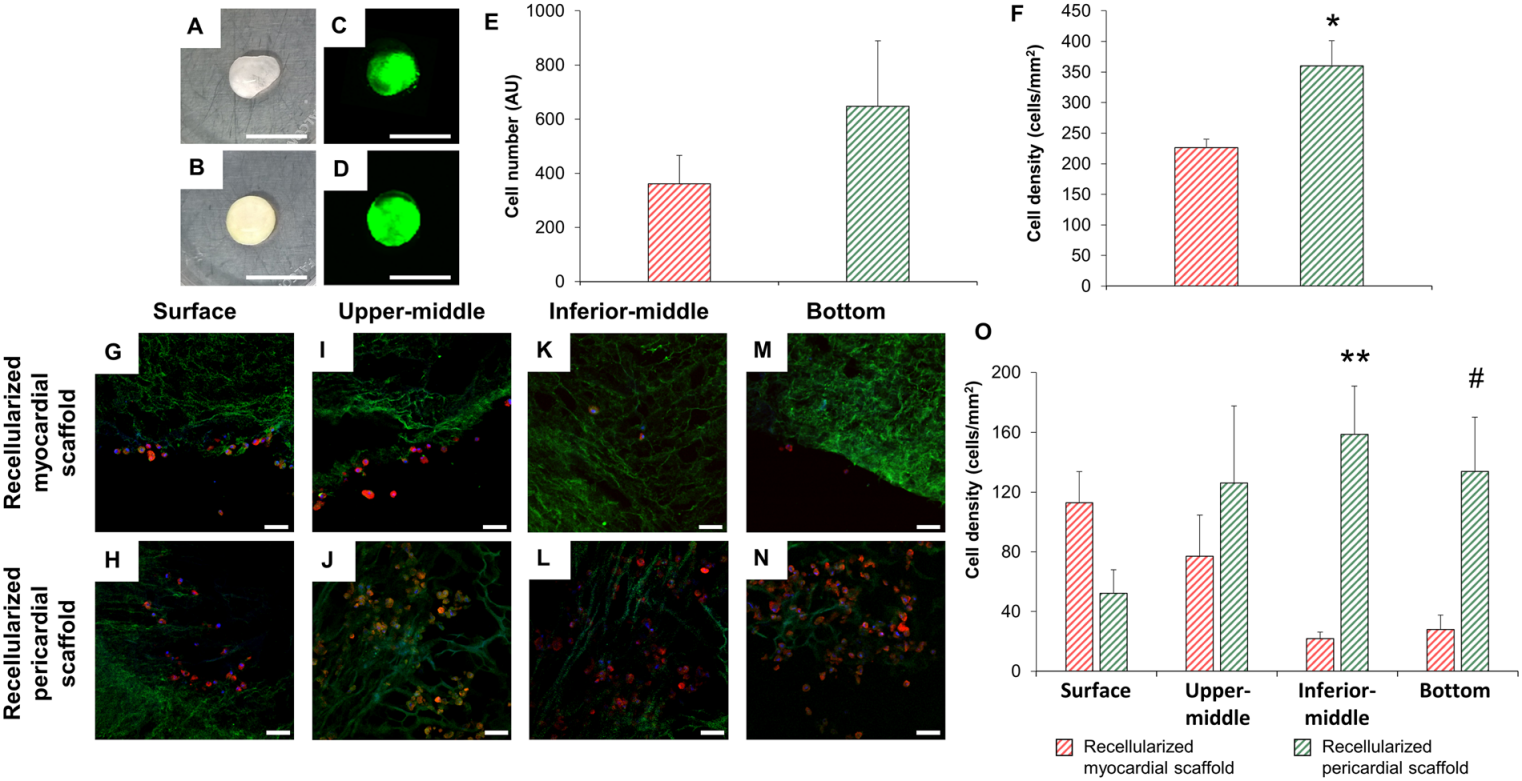
Retention of pATMSCs and scaffold penetrance after recellularization. Photographs of the decellularized (**A**) myocardial and (**B**) pericardial scaffolds 2 h after cell repopulation. Representative recellularized (**C**) myocardial and (**D**) pericardial scaffolds 2 h-post recellularization showing labeled pATMSCs with NIR815 fluorescent dye (green). Scale bars = 1 cm. (**E**) Quantification of NIR815 fluorescence (arbitrary fluorescence units, AU) for both recellularized cardiac scaffolds. (**F**) Quantification of cells/mm^2^ in recellularized myocardial and pericardial scaffolds one week after recellularization. Data are reported as mean ± SEM. *P = 0.018 with Student’s t test. (**G**,**H**) Immunohistochemistry images for recellularized myocardial and pericardial scaffolds at the scaffold surface, (**I**,**J**) upper-middle, (**K**,**L**) inferior-middle, (**M**,**N**) and bottom levels, respectively. The ECM was stained with col-I (green) and pATMSCs with F-actin (red). Nuclei were counterstained with DAPI (blue). Scale bars = 50 μm. (**O**) Quantification of cell density for each scaffold depth (surface, upper-middle, inferior-middle, and bottom). Data are displayed as the mean ± SEM. **P = 0.013; ^#^*P* = 0.047 using Student’s t test.

Decellularized scaffolds maintained intrinsic organization and spatial three-dimensional distribution of the native matrix fibrils (Fig. 1A,B,G,H). Moreover, two representative matrix proteins, type-I and type-III collagens, were also properly marked, indicating preservation of matrix protein components (Fig. 1D,E,J,K). Of note, analysis of both Annexin V expression and 7-AAD staining by flow cytometry showed no significant variations in cell viability between post-thawing and following solubilization in 10% sucrose prior repopulation of scaffolds (79 ± 6% vs 75 ± 2%, respectively; *P* = 0.319) (data not shown). Upon scaffold recellularization, the presence of cells was detected using both SEM (Fig. 1C,I) and immunohistochemistry (Fig. 1F,L), confirming cell retention inside the scaffolds one week after repopulation. Two-hours post-recellularization, visual examination of the scaffolds suggested a more regular retention of the cell-hydrogel mixture for the pericardial scaffold, with no liquid leakage observed (Fig. 2A,B). However, cell quantification did not reveal significant differences between the scaffolds (Fig. 2C–E).

**Figure 3:**
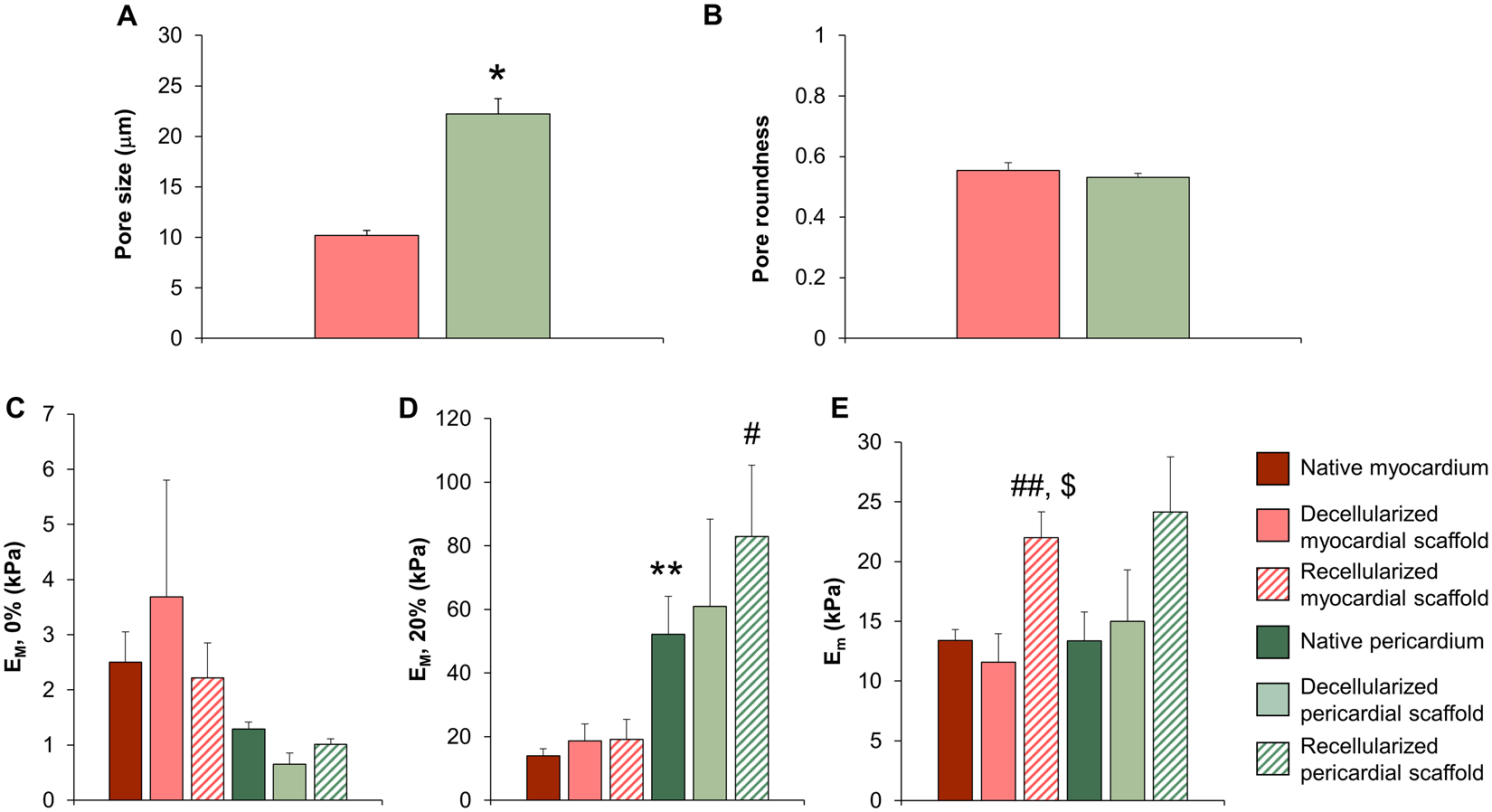
Ultrastructure, macroscopic, and microscopic mechanical characterization. (**A**) Pore size and (**B**) pore roundness measurements in both myocardial and pericardial scaffolds. *P = 0.002 with Student’s t test. (**C**) Macroscopic stiffness (*E*_*M*_) of myocardium and pericardium strips measured at the unstretched length (**D**) and 20% stretch using tensile testing in native tissues and decellularized and recellularized scaffolds. (**E**) Microscopic stiffness (*E*_*m*_) of myocardium and pericardium 50 μm thick slices measured with AFM, for the same tissue conditions. Data are presented as the mean ± SEM. **P < 0.001 between native pericardium and native myocardium. ^#^*P* = 0.044 between recellularized pericardium and recellularized myocardium, using Student’s t test. ^##^*P* = 0.013 and ^$^*P* = 0.005 between recellularized myocardium vs. native and decellularized myocardium, respectively, with ANOVA test with Tukey’s *post*-*hoc* correction for multiple comparisons analysis.

One week post-recellularization, cell retention was significantly higher for pericardial scaffolds (226 ± 14 vs. 360 ± 41 cells/mm^2^; *P* = 0.018) (Fig. 2F). Cell distribution, penetrance, and retention through the scaffold thickness differed between the scaffolds, with a superficial cellular distribution and limited cell migration in myocardial matrix and a uniform distribution with complete migration of pATMSCs throughout the complete scaffold thickness in pericardial matrix (Fig. 2G–N) (Surface: 113 ± 21 vs. 52 ± 16 cells/mm^2^; *P* = 0.082; upper-middle: 77 ± 28 vs. 126 ± 52 cells/mm^2^; *P* = 0.449; inferior-middle: 22 ± 5 vs. 159 ± 32 cells/mm^2^; *P* = 0.013; bottom: 28 ± 10 vs. 134 ± 36 cells/mm^2^; *P* = 0.047, for recellularized myocardial and pericardial scaffolds, respectively) (Fig. 2O). The pore size of decellularized scaffolds was significantly higher in pericardial scaffolds (10.2 ± 0.5 vs. 22.2 ± 1.5 μm; *P* = 0.002; Fig. 3A), although, no difference in pore roundness was observed between decellularized myocardial and pericardial scaffolds (Fig. 3B).

The mechanical properties of cardiac tissue were evaluated at two different scales: macromechanics by tensile testing and micromechanics using AFM. Macroscopic stiffness of the native myocardium was 2.5 ± 0.5 kPa at unstretched length and increased to 14.0 ± 2.3 kPa at 20% stretch (Fig. 3C,D). No significant differences were found after decellularization and recellularization. The non-linear parameter *α* (Equation 6) was 13.1 ± 0.7 in the native myocardial tissue with no significant changes in the decellularized and recellularized scaffolds. Tensile tests did not reveal significant differences between native, decellularized, and recellularized samples neither at unstretched length (Fig. 3C) nor at 20% stretch (Fig. 3D). Interestingly, macroscopic stiffness did not change significantly when the acellular myocardial scaffold was lyophilized, sterilized, or embedded in RAD16-I hydrogel (Supplementary Figure S1A,B). Microscopic stiffness measured in the native myocardium with AFM (13.4 ± 0.9 kPa) was ∼5-fold higher than macroscopic stiffness found in unstretched strips. Similar to the macroscopic mechanical behavior, no significant changes were observed between decellularized, lyophilized, sterilized, or rehydrated myocardial scaffolds (Supplementary Figure S1C). In contrast to macroscopic measurements, microscopic stiffness of recellularized scaffolds (22.0 ± 2.2 kPa) was ∼2-fold stiffer than native tissue (13.4 ± 0.9 kPa) and decellularized scaffolds (11.6 ± 2.4 kPa) (respectively-test *P* < 0.05) (Fig. 3E).

There were no significant changes in macroscopic and microscopic stiffness between native, decellularized and recellularized pericardium (Fig. 3C–E). No significant differences were found in the unstretched length between pericardium and myocardium. However, native pericardial tissue and its recellularized scaffolds were ∼3-fold stiffer than the myocardium (Student t test *P* < 0.001 and *P* < 0.05, respectively) (Fig. 3D). Consistently, the non-linear parameter α (Equation 6) of the native (36.9 ± 4.5), decellularized (31.2 ± 3.6) and recellularized (23 ± 1.8) pericardium was ∼2 fold higher (*P* < 0.05) than the myocardium, reflecting a stronger strain-hardening behavior. In agreement with myocardium mechanical behavior, no significant changes were found when comparing data along the different recellularization steps (Supplementary Figure S1A–C).

### Proteomic characterization

**Figure 4:**
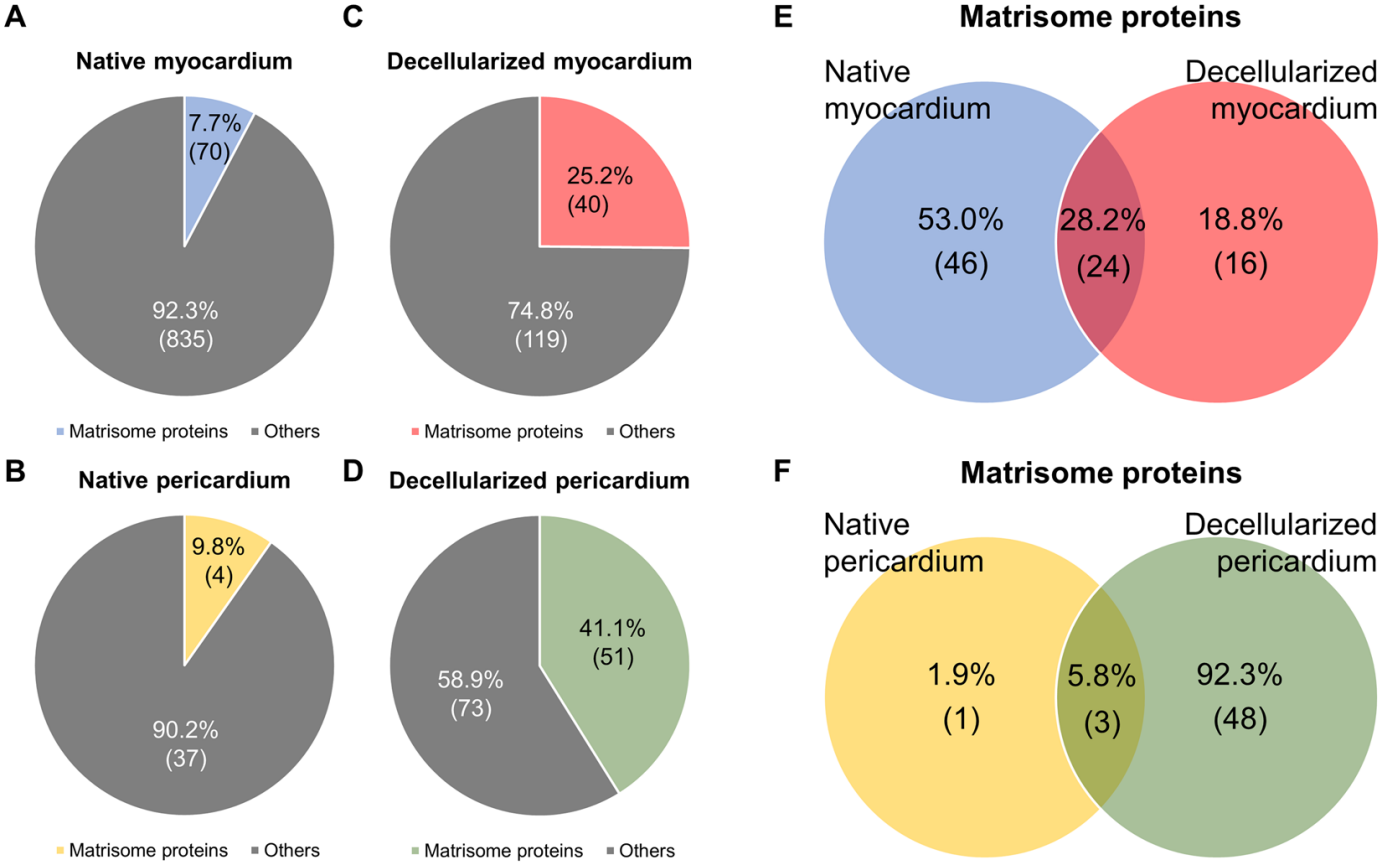
Protein content of decellularized cardiac scaffolds. Number of matrisome and other proteins identified in (**A**) the native myocardium (reported by Guyette *et al*.^16^) and (**B**) in the native pericardium (collected by Griffiths *et al*.^17^). Total protein content, classified as matrisome and other proteins, for (**C**) the decellularized myocardial and (**D**) decellularized pericardial scaffolds. (**E**) Venn diagram displaying the unique proteins identified in the native or decellularized myocardium, and their common proteins. (**F**) Venn diagram indicating the number of unique and shared proteins between the native and decellularized pericardia. Results are represented as the percentage of the total protein number, and in brackets, as the number of proteins for each classification.

Decellularization of both cardiac tissues was associated with enrichment of matrisome/ECM-related proteins, with 7.7% and 9.8% of proteins in native myocardium and pericardium, respectively, and 25.2% and 41.1% in decellularized myocardial and pericardial scaffolds, respectively (Fig. 4A–D). For decellularized myocardium matrisome proteins, 24 of the 40 proteins were also in the native tissue, and 16 proteins were detected exclusively within the decellularized myocardium. In the pericardium, matrisome enrichment was more pronounced: only three proteins were common in the decellularized and native pericardia; while forty-eight proteins were only detected in the decellularized pericardium and there was just one unique protein in the native pericardium (Fig. 4E,F).

**Figure 5:**
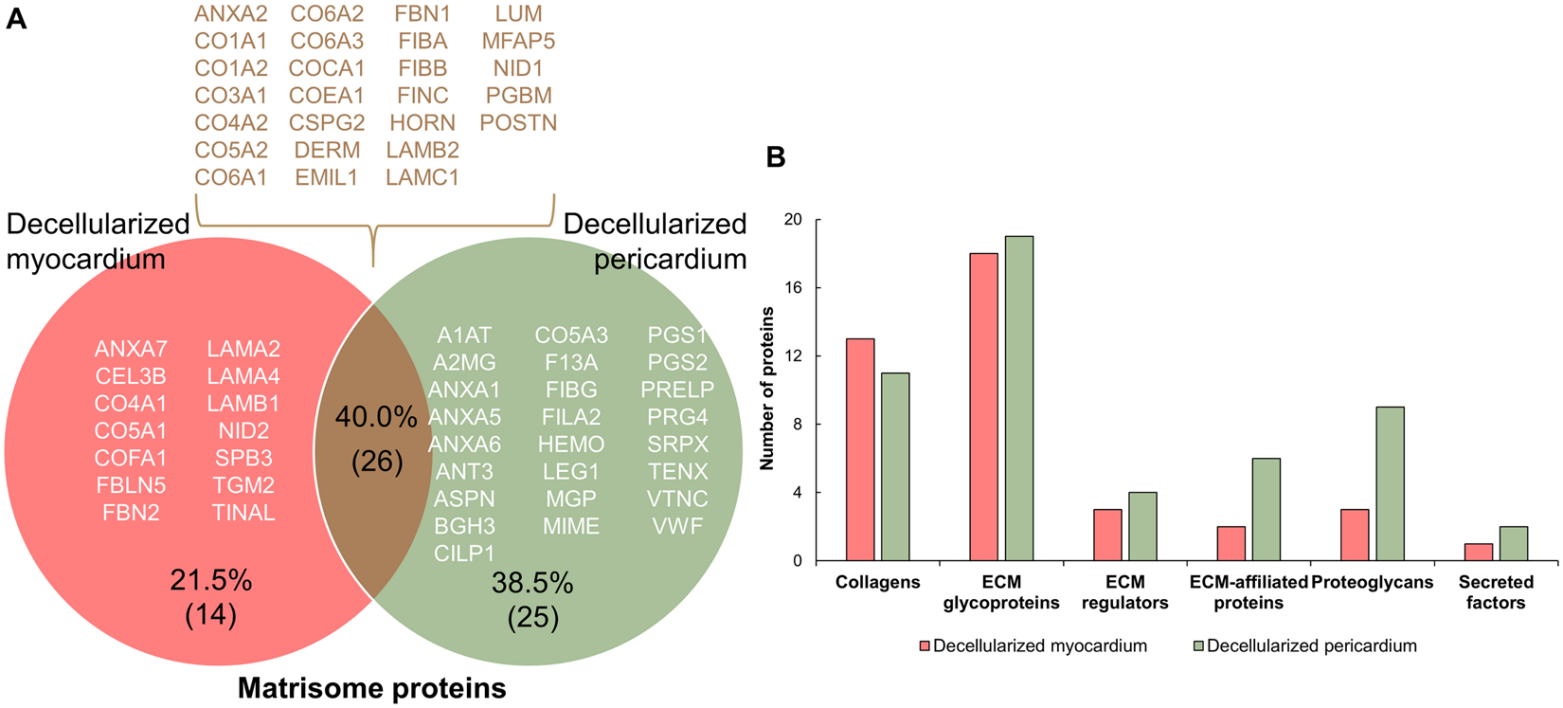
Comparison of matrisome proteins in decellularized myocardial and pericardial scaffolds. (**A**) Individual and common proteins of the decellularized myocardial and pericardial scaffolds. Data are represented as a relative percentage of the total number of proteins and, within brackets, the number of proteins for each classification. The UniProt accession name for the detected proteins is provided for each case. (**B**) Number of proteins for the decellularized myocardial and pericardial scaffolds in each of the following matrisome protein subdivisions: collagens, ECM glycoproteins, ECM regulators, ECM-affiliated proteins, proteoglycans, and secreted factors.

Comparative analysis of the generated acellular myocardial and pericardial tissues revealed they shared 40% of the matrisome proteins. Among them, the most remarkable proteins were the different collagen subtypes, responsible for maintaining the ECM structure and modulating cell differentiation; the laminin family and heparan sulfate, mainly involved in cell adhesion; and fibronectin, which is involved in different cellular processes such as adhesion, survival, and differentiation. In addition, the number of unique proteins identified was notably higher for the pericardium, with up to 25 distinctive matrisome proteins vs. just 14 in the myocardium (Fig. 5A and Tables 1–3). Finally, subdivision of the ECM-proteins showed more proteins within each ECM protein subtype for the decellularized pericardium, except for the collagen subclass (Fig. 5B).

**Table 1:** Common proteins shared by decellularized myocardial and pericardial scaffolds.

Accession	Protein	Score	Category
ANXA2	Annexin A2	59	ECM-affiliated Proteins
CO1A1	Collagen alpha-1(I) chain	89.6	Collagens
CO1A2	Collagen alpha-2(I) chain	46.6	Collagens
CO3A1	Collagen alpha-1(III) chain	89.3	Collagens
CO4A2	Collagen alpha-2(IV) chain	74.8	Collagens
CO5A2	Collagen alpha-2(V) chain	49.7	Collagens
CO6A1	Collagen alpha-1(VI) chain	471	Collagens
CO6A2	Collagen alpha-2(VI) chain	401.7	Collagens
CO6A3	Collagen alpha-3(VI) chain	765.2	Collagens
COCA1	Collagen alpha-1(XII) chain	41.6	Collagens
COEA1	Collagen alpha-1(XIV) chain	149.1	Collagens
CSPG2	Versican core protein	62.5	Proteoglycans
DERM	Dermatopontin	72.5	ECM Glycoproteins
EMIL1	EMILIN-1	159.7	ECM Glycoproteins
FBN1	Fibrillin-1	1734	ECM Glycoproteins
FIBA	Fibrinogen alpha chain	42.1	ECM Glycoproteins
FIBB	Fibrinogen beta chain	75.1	ECM Glycoproteins
FINC	Fibronectin	485.2	ECM Glycoproteins
HORN	Hornerin	58.4	Secreted Factors
LAMB2	Laminin subunit beta-2	307.8	ECM Glycoproteins
LAMC1	Laminin subunit gamma-1	674.8	ECM Glycoproteins
LUM	Lumican	146.2	Proteoglycans
MFAP5	Microfibrillar-associated protein 5	69.2	ECM Glycoproteins
NID1	Nidogen-1	107.4	ECM Glycoproteins
PGBM	Basement membrane-specific heparan sulfate proteoglycan core protein	696.8	Proteoglycans
POSTN	Periostin	68.5	ECM Glycoproteins

List of the common proteins (26) found in both decellularized cardiac tissues, with their UniProt accession name, protein name, identification score, and matrisome category classification indicated.

**Table 2:** Unique proteins identified for the decellularized myocardial scaffolds.

Accession	Protein	Score	Category
ANXA7	Annexin A7	68.7	ECM-affiliated Proteins
CEL3B	Chymotrypsin-like elastase family member 3B	51.7	ECM Regulators
CO4A1	Collagen alpha-1(IV) chain	60.9	Collagens
CO5A1	Collagen alpha-1(V) chain	46.4	Collagens
COFA1	Collagen alpha-1(XV) chain	98.6	Collagens
FBLN5	Fibulin-5	58.1	ECM Glycoproteins
FBN2	Fibrillin-2	170.7	ECM Glycoproteins
LAMA2	Laminin subunit alpha-2	145.3	ECM Glycoproteins
LAMA4	Laminin subunit alpha-4	186	ECM Glycoproteins
LAMB1	Laminin subunit beta-1	86.8	ECM Glycoproteins
NID2	Nidogen-2	98.6	ECM Glycoproteins
SPB3	Serpin B3	40	ECM Regulators
TGM2	Protein-glutamine gamma-glutamyltransferase 2	173.2	ECM Regulators
TINAL	Tubulointerstitial nephritis antigen-like	131.1	ECM Glycoproteins

List of the unique proteins (14) detected for the acellular myocardial scaffolds, with their UniProt accession name, protein name, identification score, and matrisome category indicated.

**Table 3:** Unique proteins detected in the decellularized pericardial scaffolds.

Accession	Protein	Score	Category
A1AT	Alpha-1-antitrypsin	64.3	ECM Regulators
A2MG	Alpha-2-macroglobulin	208.8	ECM Regulators
ANT3	Antithrombin-III	46.6	ECM Regulators
ANXA1	Annexin A1	244.4	ECM-affiliated Proteins
ANXA5	Annexin A5	301.9	ECM-affiliated Proteins
ANXA6	Annexin A6	101.1	ECM-affiliated Proteins
ASPN	Asporin	48.2	Proteoglycans
BGH3	Transforming growth factor-beta-induced protein ig-h3	233.6	ECM Glycoproteins
CILP1	Cartilage intermediate layer protein 1	49.5	ECM Glycoproteins
CO5A3	Collagen alpha-3(V) chain	49.3	Collagens
F13A	Coagulation factor XIII A chain	86.2	ECM Regulators
FIBG	Fibrinogen gamma chain	1172.9	ECM Glycoproteins
FILA2	Filaggrin-2	49.8	Secreted Factors
HEMO	Hemopexin	54.7	ECM-affiliated Proteins
LEG1	Galectin-1	143.1	ECM-affiliated Proteins
MGP	Matrix Gla protein	60.8	ECM Glycoproteins
MIME	Mimecan	125.8	Proteoglycans
PGS1	Biglycan	193.4	Proteoglycans
PGS2	Decorin	212.3	Proteoglycans
PRELP	Prolargin	151.2	Proteoglycans
PRG4	Proteoglycan 4	83.7	Proteoglycans
SRPX	Sushi repeat-containing protein SRPX	72.5	ECM Glycoproteins
TENX	Tenascin-X	56.2	ECM Glycoproteins
VTNC	Vitronectin	229.2	ECM Glycoproteins
VWF	von Willebrand factor	135.5	ECM Glycoproteins

The unique proteins (25) identified in the decellularized pericardial scaffold, with the UniProt accession name, protein name, identification score, and matrisome category indicated.

### Animal Experimentation

Four animals died during MI induction due to ventricular fibrillation and 4 were excluded from the study after post-operative infections. Thus, 66 animals were included in the experimental protocol in the Per-MI (n = 15), Myo-MI (n = 7), Per-ATMSCs (n = 20), Myo-ATMSCs (n = 7), and Control-MI (n = 17) groups.

### Cardiac Function Assessment

**Figure 6:**
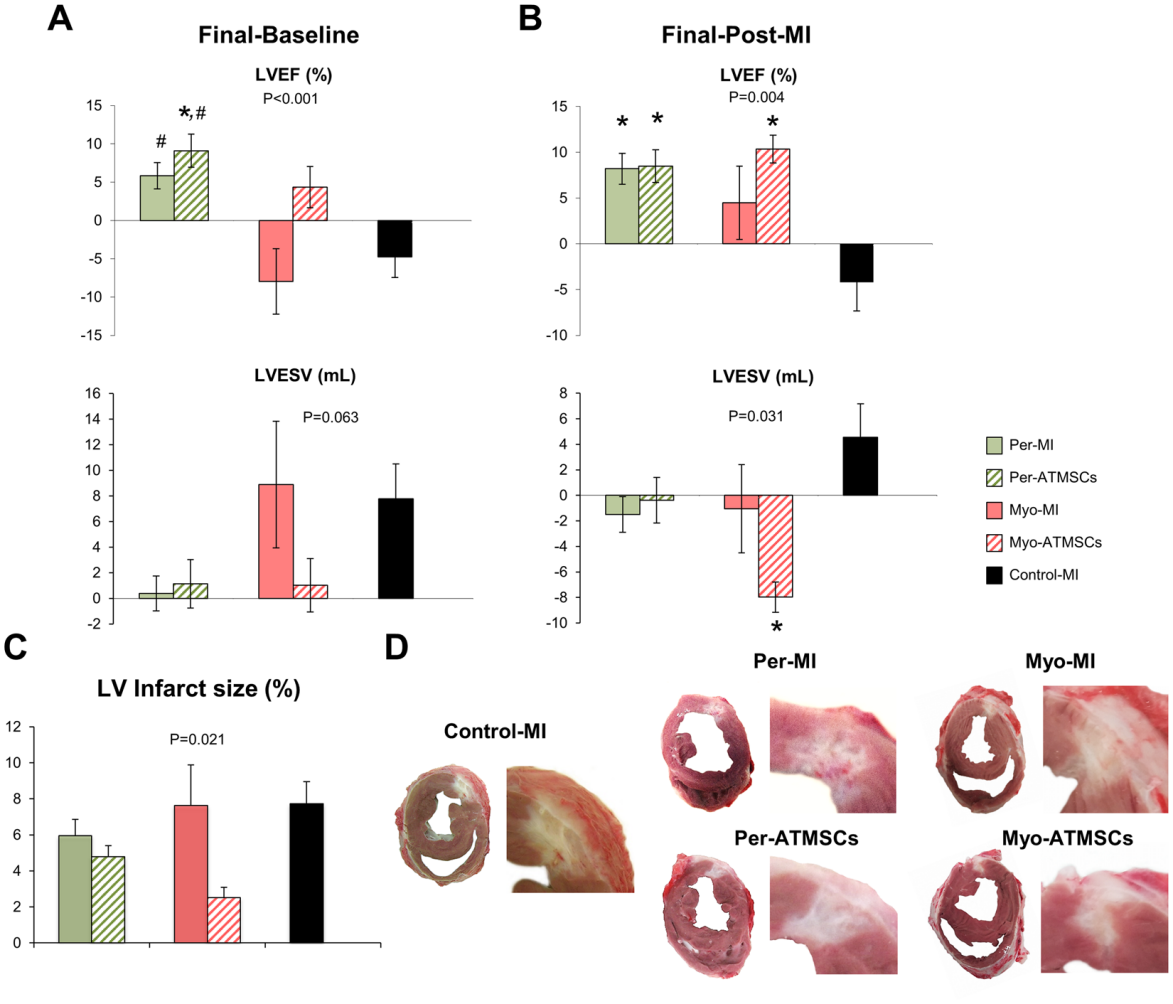
Cardiac function and infarct morphometric analysis. Histograms of the differentials between 40 days of follow-up and (**A**) baseline or (**B**) post-MI among the Per-MI, Per-ATMSCs, Myo-MI, Myo-ATMSCs, and Control-MI groups for LVEF and LVESV. *P < 0.05 vs. control-MI; ^#^*P* < 0.05 vs. Myo-MI. (**C**) Percentage of the LV infarct area measured in all heart sections for all study groups after 40 days of follow-up (*P < 0.05 vs. control-MI); and (**D**) representative heart sections for each study group showing the infarcted area of the LV. Statistical analysis was performed using ANOVA test with Tukey’s *post*-*hoc* correction for multiple comparisons.

Cardiac function parameters measured for all animal groups (LVEF, LVEDV and LVESV) at different time points have been summarized in Supplementary Table S1. The LVEF changes between sacrifice and baseline indicated improved function for Per-MI and Per-ATMSCs (*P* < 0.001). Changes in the LVESV showed a trend towards an improvement in the studied groups (Fig. 6A). Similarly, LVEF changes between sacrifice and post-MI were also significant: (*P* = 0.004). In addition, LVESV changes between sacrifice and post-MI were significant (*P* = 0.031) (Fig. 6B). Changes in the absolute values at 40 days for LVEF (*P* = 0.017) and LVESV (*P* = 0.020) were also significant.

### Infarct size

The LV infarct size was statistically different between the Per-MI (n = 13), Myo-MI (n = 7), Per-ATMSCs (n = 18), Myo-ATMSCs (n = 7), and Control-MI (n = 10) animals (5.9 ± 0.9 vs. 7.6 ± 2.3 vs. 4.8 ± 0.6 vs. 2.5 ± 0.6 vs. 7.7 ± 1.2%, respectively; *P* = 0.021) (Fig. 6C,D).

### Immunohistological analysis

**Figure 7:**
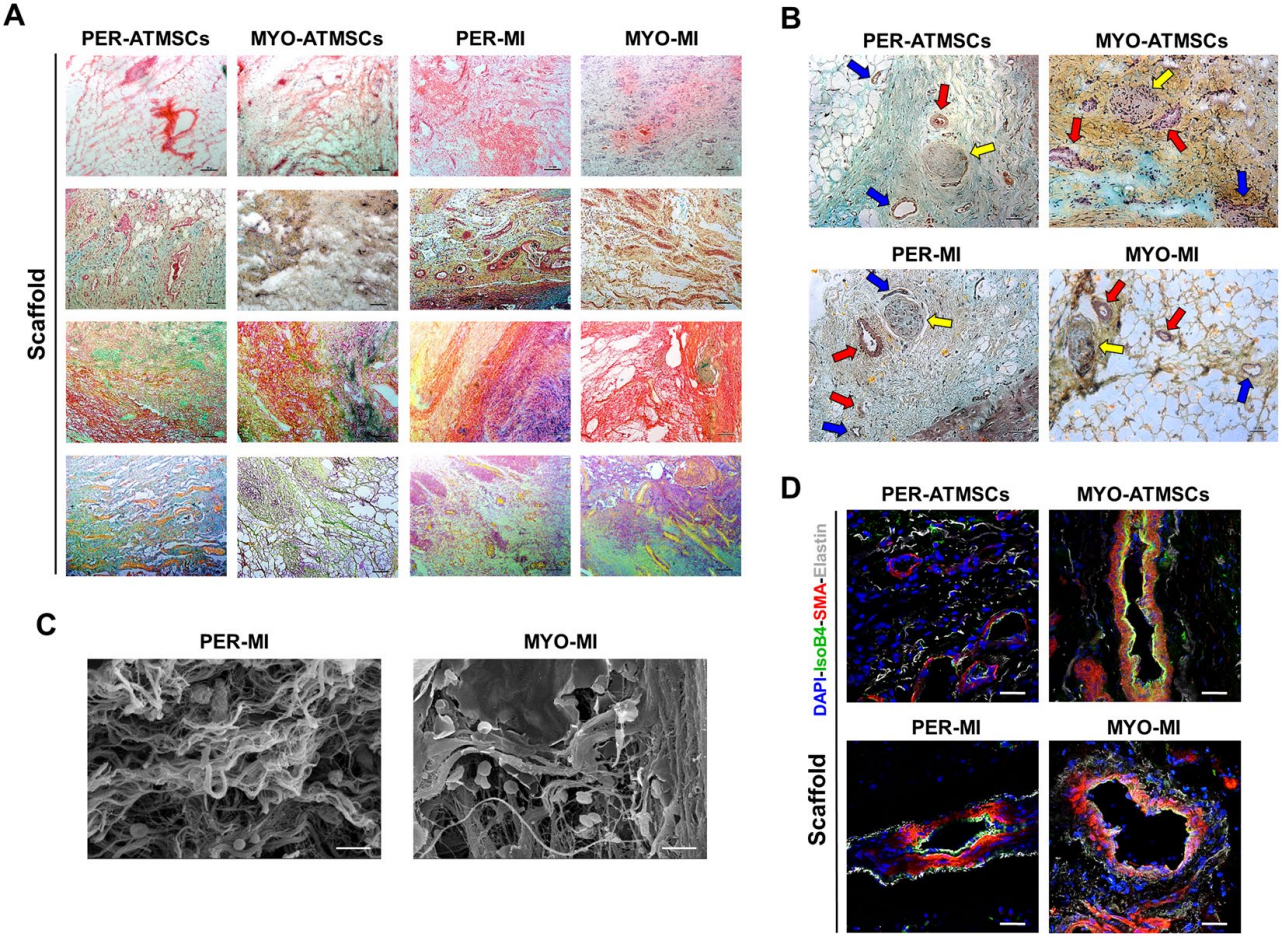
Histological and immunohistochemical analysis after *in vivo* scaffold implantation. (**A**) Representative images (top to bottom) of H/E, Movat’s pentachrome, Movat’s pentachrome for simultaneous collagen and mucopolysaccharide acid staining and Gallego’s modified trichrome. Scale bar = 100 μm. (**B**) Movat’s pentachrome of the scaffolds from the Per-ATMSCs, Myo-ATMSCs, Per-MI, and Myo-MI groups displaying the presence of arterial blood vessels (red arrows), veins (blue arrows), and nerve fibers (yellow arrows). Scale bar = 50 μm. (**C**) Representative SEM images of scaffolds from Per-MI and Myo-MI experimental groups after sacrifice. (**D**) Immunohistochemical images of the scaffolds against IsoB4 (green), SMA (red), and elastin (white) antibodies confirming the presence of arteriolar blood vessels positive for SMA. Nuclei appear counterstained in blue. Scale bars = 50 μm.

Correct adhesion of the implanted graft with subjacent myocardium was observed in all animals after sacrifice. After histological analysis, Movat’s modified staining confirmed the collagen composition of the scaffolds (red), the presence of sulfated mucopolysaccharides (violet), red blood cells (yellow) as well as lymphocytes and plasma cells (green). Gallego’s modified staining also confirmed the presence of collagen in scaffolds (brilliant green), elastic fibers (fuchsine violet) and squamous epithelium (yellow) (Fig. 7A). Moreover, scaffolds of all experimental groups showed functional blood vessels connected to host vascularization, which was confirmed by the presence of erythrocytes in the lumen, some even with SMA in the medial layer (Fig. 7A,B,D), and newly formed nerves within the scaffolds (Fig. 7B).

## Discussion

Cardiac tissue engineering is mainly based on the combination of a biomimetic scaffold with a cell lineage of interest. Here, we characterized the structure as well as the macro and micromechanical scaffold properties after decellularization and recellularization of two biological scaffolds from myocardial and pericardial tissue. Importantly, no adverse events were associated with pericardium isolation from patients, following a safe cardiac surgery procedure previously reported by our group^13,18,19^. We also evaluated their proteome composition and the ability of the two engineered grafts (once recellularized with ATMSCs) to restore cardiac function.

When using an acellular framework for the scaffold design, the ECM inherent properties, such as structure, mechanics, and protein composition, must be ideally maintained from the beginning. Matrix disruption due to decellularization agents must be minimized to recreate the native physiological milieu, as structure preservation has been related to cell differentiation, migration, and alignment^7,8^. Mechanical stiffness modulates cell adhesion, differentiation, proliferation, and migration^4,6,20^, as well as contractility^21^. We have performed a multiscale study of myocardial and pericardial mechanics. Stiffness measured at the macroscale is important to assess the mechanical matching between the ventricular wall and the attached patch. The similar macroscopic stiffness of the native tissue and its recellularized scaffold indicates homogeneous deformation of the patch and healthy ventricular wall during heart beating. On the other hand, stiffness measured with AFM characterizes the mechanical properties at the microscale, which is the scale at which cells sense their mechanical microenvironment. AFM probes micromechanics of the sample under unstretched conditions. Therefore, the ~5-fold higher stiffness we found with AFM (Fig. 3E) as compared to that measured at 0% stretch with tensile assays (Fig. 3C) suggests that cells sense a local niche substantially stiffer than the stiffness of the whole 3D matrix scaffold. This differential stiffness indicates that scaffold macromechanics is determined by the intrinsic micromechanical properties of the ECM as well as by the 3D topology of the matrix. Although no significant differences were found in macroscale stiffness at 0% stretch between myocardial and pericardial scaffolds (Fig. 3C), the more marked strain-hardening behavior exhibited by the pericardium (~2-fold higher α) resulted in a ~2-fold stiffer pericardial scaffold at 20% stretch (Fig. 3D). It should be noted that ventricular tissues are subjected to ~20% stretch during heart beating indicating that the decellularized pericardial tissue provides a stiffer scaffold under physiological conditions. According to our results, macro and micromechanics were well-maintained in both cardiac scaffolds following decellularization. No significant mechanical changes were observed after subjecting the decellularized scaffolds to lyophilization, sterilization and hydrogel embedment. This allows the conservation and storage of the decellularized scaffolds until later use. Of note, the matrix pore size was significantly larger in pericardial scaffolds, which correlated with the findings from a cardiomyocyte transverse section reported in a previous study^22^. Despite the addition of ATMSCs significantly increased myocardial micromechanics, the resulting stiffness for both recellularized myocardial and pericardial scaffolds was within the optimal range (10–20 kPa) to drive physiological processes such as cardiomyocyte maturation, contraction, and cardiac lineage commitment, as reported in the literature^23,24^. Therefore, seeding ATMSCs on top of the decellularized myocardium did not have a major impact on the matrix micromechanics; rather, it resulted in an overall preservation of the scaffold structure and mechanics following the recellularization process.

Regarding protein content, the native matrisome proteins were preserved in both cardiac tissues upon decellularization, which is consistent with prior studies^16^, and some of the main cardiac ECM components were identified, including: collagens (type-I, -III, -IV, -V, -VI), laminin, emilin-1, fibronectin, lumican, heparan sulfate proteoglycan, nidogen, and periostin. These key proteins act as mediators or effectors of many cellular processes, resulting in favored ATMSC adhesion and survival. Indeed, laminins, fibronectin, and type-I and IV collagens enhance cellular survival^25,26^; whereas, fibronectin^27^, lumican^28^, and type-IV and -VI collagens^29,30^ are associated with cell attachment through specific interactions with integrin cell receptors. Galectin-1, vitronectin, decorin, and tenascin-X are involved in cell adhesion^27,31,32^ and were also identified in our decellularized pericardial scaffolds. This finding, along with the optimal pore diameter suitable for cell infiltration^22,33^, may explain the increased cell retention and penetrance observed in these scaffolds.

According to the 3 R principle (Replacement, Reduction and Refinement), we included previous animal experimentation models^13,19,34^ in this work, performed by the same surgical team under identical conditions for comparison. We demonstrated that the implantation of the engineered cardiac grafts led to improvements in LVEF and/or LVESV, limited infarct size expansion, and coupling with the underlying myocardium, as indicated by the vascularization and innervation of the grafts. The benefits exhibited here by both recellularized scaffolds are greater than those reported for direct intracoronary or intramyocardial injection of ATMSCs^35^, or administration of an acellular myocardial^36^ or pericardial scaffold^37^. The combination of the cardiac scaffolds with ATMSCs showed more improvement than the non-recellularized scaffolds, indicating a synergistic effect, as reported previously^38^. Scaffolds may be beneficial for triggering MI salvage, by providing a favorable microenvironment for the seeded ATMSCs, and for recruiting endogenous stem cells towards the infarct bed^39^, boosting both vascularization and cardiomyogenesis. The contribution of ATMSCs to MI recovery has also been previously described, and the most recent evidence points towards paracrine signaling, rather than direct effects of ATMSCs^40^. Paracrine action would be consistent with a low number of retained or seeded cells, as in our study, being able to promote effects^41^, such as vessel formation, protecting resident cardiomyocytes from apoptosis, and mobilizing resident stem cells to potentiate vascularization and cardiomyogenesis^42^. Remarkably, in the present work, both cell-free and ATMSC-recellularized cardiac scaffolds, regardless of their origin, were successfully integrated in host cells, undergoing neovascularization and neoinnervation. However, further studies on ECM signaling and/or host cell recruitment pathways are required to discern which mechanisms are responsible for these processes.

## Conclusions

Two engineered cardiac grafts were successfully generated by combining ATMSCs with either myocardial or pericardial acellular scaffolds, which preserved structural framework in both cases. Nevertheless, superior preservation of mechanical intrinsic properties and higher enrichment of major cardiac ECM proteins was observed for pericardial scaffolds. Furthermore, *in vitro*, the recellularized pericardial scaffold showed higher cell retention and penetrance, and a bigger pore size compared to recellularized myocardial scaffolds. In the context of a preclinical MI model, the engineered grafts integrated with the underlying myocardium, with signs of neoinnervation and neovascularization, and improved cardiac function post-MI.

## Electronic supplementary material

Supplementary information
