# Supplementary material for: Head-to-head comparison of two engineered cardiac grafts for myocardial repair: From scaffold characterization to pre-clinical testing
Source: Sci Rep. 2018 Apr 30;8:6708. doi: 10.1038/s41598-018-25115-2 (PMC5928167; doi:10.1038/s41598-018-25115-2)
Supplement: Supplementary file 1 — Supplementary information [file 41598_2018_25115_MOESM1_ESM.docx]

**Head-to-head comparison of two engineered cardiac grafts for myocardial repair:**

**From scaffold characterization to pre-clinical testing**

Isaac Perea-Gil^1#^, Carolina Gálvez-Montón^1,2#^, Cristina Prat-Vidal^1,2,3#^, Ignasi Jorba^4,5,6^, Cristina Segú-Vergés^7^, Santiago Roura^1,2^, Carolina Soler-Botija^1,2^, Oriol Iborra-Egea^1^, Elena Revuelta-López^1^, Marco A. Fernández^8^, Ramon Farré^4,5,9^, Daniel Navajas^4,5,6^, Antoni Bayes-Genis^1,2,10,11^.

^1^ICREC Research Program, Health Science Research Institute Germans Trias i Pujol, Badalona, Spain.

^2^CIBER de Enfermedades Cardiovasculares, Madrid, Spain.

^3^Centre of Regenerative Medicine in Barcelona, Barcelona, Spain.

^4^Biophysics and Bioengineering Unit, Faculty of Medicine and Health Sciences, University of Barcelona, Barcelona, Spain.

^5^Institute for Bioengineering of Catalonia, The Barcelona Institute of Science and Technology, Barcelona, Spain.

^6^CIBER de Enfermedades Respiratorias, Madrid, Spain.

^7^Anaxomics Biotech, Barcelona, Spain.

^8^Flow Cytometry Facility, Germans Trias i Pujol Research Institute, Campus Can Ruti, Badalona, Spain.

^9^Institut d'Investigacions Biomèdiques August Pi i Sunyer, Barcelona, Spain.

^10^Cardiology Service, Germans Trias i Pujol University Hospital, Badalona, Spain.

^11^Department of Medicine, Universitat Autònoma de Barcelona, Bellaterra, Spain.

**Supplementary methods**

**Protein extraction and digestion**

From a starting amount of 100 mg of decellularized cardiac samples minced in a cold mortar, proteins were extracted using three different extraction buffer prepared in homogenizer tubes (Miltenyi Biotec, Madrid, Spain). In the first and second procedures, an urea/thiourea buffer (5M urea (Sigma-Aldrich, Madrid, Spain), 2M thiourea (Amersham Biosciences, Little Chalfont, UK), 50mM DTT (Sigma-Aldrich), 0.1% SDS), or an SDS buffer (1% SDS, 50mM TRIS HCl adjusted to pH = 6.8 (Sigma-Aldrich), 12.5% glycerol (Sigma-Aldrich), in a buffer volume (μL):sample weight (mg) relation 10:1, was added to the mashed samples and mechanically homogenized using a gentleMACS^TM^ Dissociator (Miltenyi Biotec) at 4 ^o^C. Samples were centrifuged at 2,000xg for 10 min at 4 ^o^C, both supernatant and pellet recovered and transferred to a new tube, and sonicated on ice with 3 consecutive cycles of 2 sec sonication pulses, followed by 3 sec sonication stand by, each 15 min for a total time of 1 h. The sonicated samples were centrifuged at 16,000xg for 30 min at RT, and the supernatant was collected and stored at -80 ^o^C until use. For the third process, Reagent 4 buffer (R4) (Sigma-Aldrich) was used following a buffer volume (μL):sample weight (mg) relation 1:3.3. After 4 ^o^C mechanical dissociation with the gentleMACS^TM^ Dissociator, samples were spun down at 2,000xg for 10 min at 4 ^o^C, and the total content vortexed briefly each 5 min for 45 min at RT and centrifuged at 16,000xg for 1 min at 4 ^o^C. The resulting supernatant was stored at -80 ^o^C, and the insoluble pellet was incubated with guanidine buffer (4M guanidine hydrochloride and 50mM sodium acetate (Sigma-Aldrich)) for 48 h at RT with moderate stirring at 1,000 rpm during 5 sec every 30 sec. Centrifugation at 1,500xg for 25 min at 4 ^o^C was performed, and supernatant stored at -80 ^o^C. The pellet is washed with 90% ethanol, resuspended in deglycosylation buffer (150mM sodium chloride (Sigma-Aldrich), 50mM sodium acetate, and 0.05U/mL buffer of the enzymes Chondroitinase ABC from *Proteus vulgaris*, Endo-β-galactosidase from *Bacteroides fragilis,* and Heparinase II from *Flavobacterium heparinum* (Sigma-Aldrich), and incubated 16 h at 37 ^o^C. Upon incubation completion, a centrifugation at 16,000xg for 10 min at 4 ^o^C was done, and the supernatant recovered. Finally, the stored fraction from R4 buffer, and the combined fraction from guanidine and deglyosylation buffers, were precipitated O/N at 4 ^o^C with ice-cold acetone in an acetone volume:buffer volume 5:1 ratio and subsequently dried. All buffers were supplemented with cOmplete^TM^, Mini, EDTA-free protease inhibitor cocktail (1 tablet for 10 ml of buffer) (Roche, Basel, Switzerland) and 25mM EDTA (Sigma-Aldrich) or 10mM EDTA for the deglycosylation buffer.

The extracted proteins were denatured and reduced in lithium dodecyl sulfate sample buffer (NuPAGE, Thermo Fisher, Madrid, Spain) with reducing sample agent (NuPAGE, Thermo Fisher), boiled for 10 min at 70 ^o^C, and a known quantity of protein was loaded on Bis-Tris discontinuous 4%-12% polyacrylamide gradient gels (NuPAGE, Thermo Fisher). After electrophoresis, gels were stained using SimplyBlue^TM^ SafeStain Coomassie Blue (NuPAGE, Thermo Fisher), and gel bands were excised in parallel positions across lanes and subjected to in-gel digestion by utilizing Trypsin Gold (Promega, Madison, WI). Digestion was stopped by adding trifluoroacetic acid (Sigma-Aldrich), and desalted and concentrated through a C18 SpinTips columns (Protea Biosciences, Morgantown, WV) prior to LC-MS/MS analysis.

**Peptide separation**

Peptide separation was performed on a Thermo PepMap 15-cm column with an inner diameter of 75 μm, packed with 2 μm C18 particles (Thermo Fisher) and a Thermo PepMap 2-cm pre-column with an inner diameter of 100 μm, coated with 5 μm C18 particles (Thermo Fisher). Loading solvent was H_2_O 0.1% trifluoroacetic acid at loading flow of 7 μl/minute. The chromatographic gradient increased from 1% B to 5% B during 5 min; from 5 to 61 min B phase raised to 99 %; and after that, at 62 min, % B phase was 1%, until minute 72 to equilibrate the column; where solvent A was H_2_O 0.1% formic acid; and solvent B was acetonitrile with 0.1% formic acid, and the flow rate was 0.5 μl/min. The column oven operated at 35 ^o^C.

**Cardiac ATMSCs labeling for *in vitro* scaffold cell tracking**

Before cell seeding over decellularized scaffolds, cardiac ATMSCs were labeled with the CellVue® NIR815 cell labeling kit (LI-COR Biosciences, Inc., Lincoln, NE) following manufacturer instructions. The near-infrared fluorescent dye NIR815 was detected and quantified at a wave length of 800 nm by using the Odyssey® CLx Imaging system (LI-COR).

**Cell penetrance and density quantification**

For cellular penetrance and migration, total number of cell nuclei was quantified using ZenBlue software (Zeiss) in recellularized scaffolds two-hours post-reseeding (n=3), at different scaffold depth thickness: surface (from 0 to 25% of total thickness); upper-middle (from 0% to 25% of total thickness); inferior-middle (from 25% to 50% of total thickness); and bottom (from 75% to 100% of total thickness). Cell retention one week after recellularization was also quantified using nuclei recount for both cardiac recellularized scaffolds (n=4 and 3 for recellularized myocardial and pericardial scaffolds, respectively) using ZenBlue software (Zeiss). At least 5 visual fields were counted for each sample.


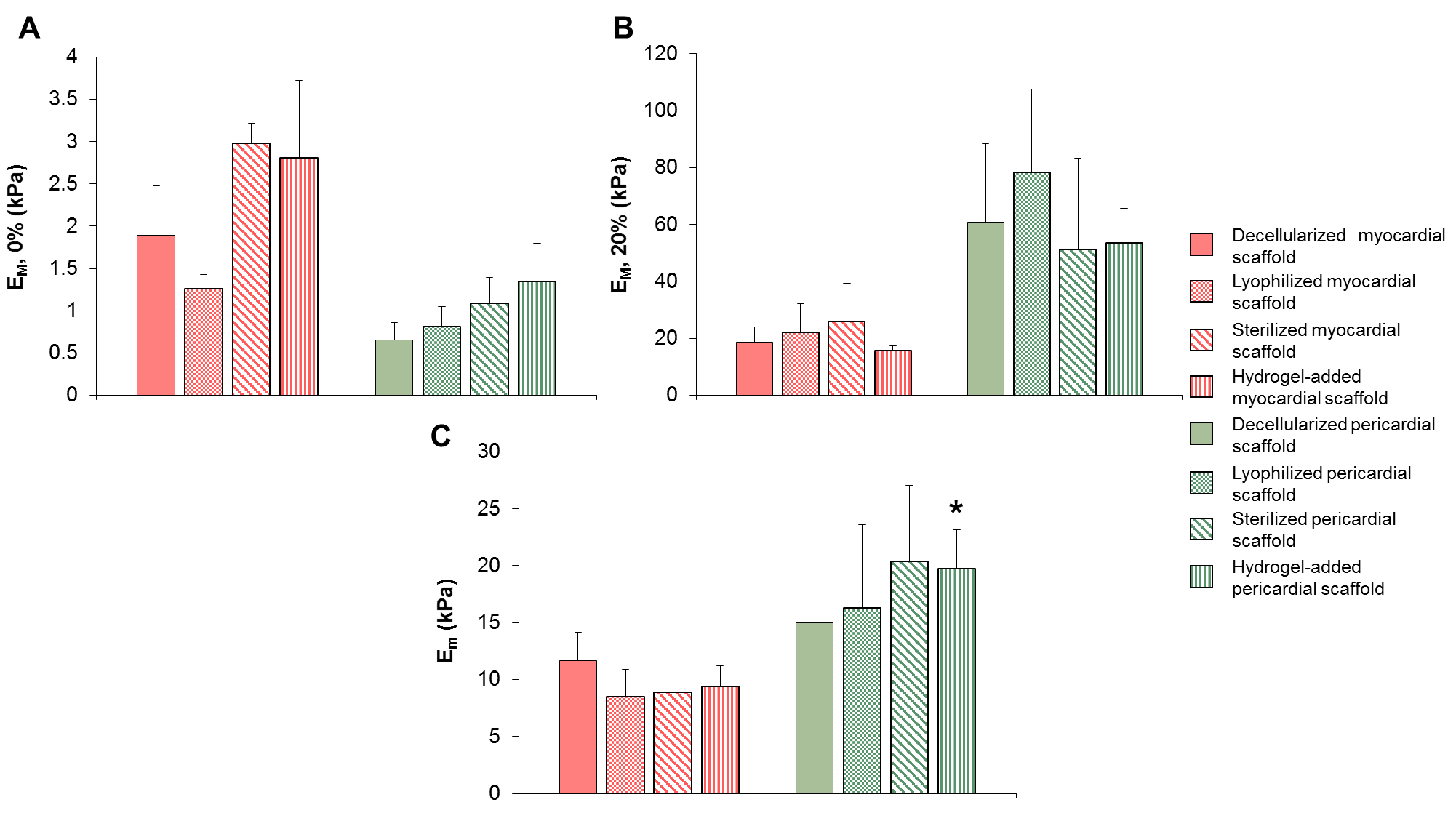


**Supplementary Figure S1.** Macro and micromechanics of different stages between decellularized and recellularized cardiac scaffolds. (A) Macroscopic stiffness (EM) of myocardium and pericardium strips measured at the unstretched length and (B) 20% stretch, using tensile testing for each of the following conditions: decellularized, lyophilized, sterilized with gamma irradiation, and hydrogel-refilled scaffolds. (C) Micromechanical stiffness (Em) measured with AFM for the same tissue conditions *P=0.021 hydrogel-refilled pericardial scaffold vs. hydrogel-refilled myocardial scaffold, with Student’s t test. Data are indicated as mean±SEM.

**Supplementary Table S1. MRI cardiac function parameters.** LVEF, LVEDV and LVESV parameters measured for the five experimental groups (Per-MI, Myo-MI, Per-ATMSCs, Myo-ATMSCs and Control-MI) at each timepoint: baseline, post-MI and final. Values are shown as mean ± SEM. LVEF= left ventricular ejection fraction; LVEDV= left ventricular end-diastolic volume; LVESV= left ventricular end-systolic volume.

|  | LVEF_Baseline_ | LVEF_Post-MI_ | LVEF_Final_ | LVEDV_Baseline_ | LVEDV_Post-MI_ | LVEDV_Final_ | LVESV_Baseline_ | LVESV_Post-MI_ | LVESV_Final_ |
| --- | --- | --- | --- | --- | --- | --- | --- | --- | --- |
| **Per-MI** | 54.8 ± 1.6 | 52.5 ± 1.5 | 60.7 ± 1.5 | 46.78 ± 1.93 | 48.71 ± 1.87 | 55.19 ± 2.77 | 21.20 ± 1.17 | 23.07 ± 1.04 | 21.58 ± 1.21 |
| **Myo-MI** | 58.8 ± 2.6 | 48.4 ± 3.0 | 52.9 ± 2.2 | 63.71 ± 6.31 | 64.74 ± 5.51 | 66.97 ± 5.11 | 26.45 ± 3.37 | 33.30 ± 3.25 | 32.25 ± 3.59 |
| **Per-ATMSCs** | 51.4 ± 1.6 | 52.0 ± 6.1 | 60.5 ± 2.4 | 47.53 ± 2.34 | 51.77 ± 2.46 | 60.80 ± 3.47 | 23.50 ± 1.62 | 25.01 ± 1.51 | 24.63 ± 2.56 |
| **Myo-ATMSCs** | 58.0 ± 2.2 | 50.8 ± 2.6 | 61.3 ± 3.1 | 55.78 ± 3.11 | 66.21 ± 2.71 | 62.91 ± 2.61 | 23.71 ± 2.40 | 32.78 ± 2.48 | 24.73 ± 2.82 |
| **Control-MI** | 55.3 ± 0.9 | 54.8 ± 1.8 | 50.6 ± 2.3 | 54.88 ± 3.78 | 61.40 ± 3.96 | 65.59 ± 2.00 | 24.47 ± 1.93 | 27.68 ± 1.98 | 32.26 ± 1.18 |
